# Uric acid is associated with increased risk of myocardial infarction: results from NHANES 2009-2018 and bidirectional two-sample Mendelian randomization analysis

**DOI:** 10.3389/fendo.2024.1424070

**Published:** 2024-10-18

**Authors:** Ting Deng, Xiaoying Liu

**Affiliations:** ^1^ Department of General Practice, The First Affiliated Hospital of Dalian Medical University, Dalian, China; ^2^ Cadre Health Center, The First People’s Hospital of Aksu Prefecture of Xinjiang, Aksu, China

**Keywords:** uric acid, myocardial infarction, NHANES, cross-sectional study, Mendelian randomization

## Abstract

**Aim:**

Although a growing number of studies have shown that elevated uric acid (UA) levels are associated with multiple cardiovascular risk factors and progression of coronary artery disease, the causal relationship between UA and the occurrence of myocardial infarction (MI) remains uncertain. The aim of this study was to investigate the relationship between UA and the risk of MI.

**Methods:**

We screened 23,080 patients in the National Health and Nutrition Examination Survey (NHANES) database for 2009-2018 and explored the association between UA and MI risk using multivariate logistic regression model. In addition, a two-way two-sample Mendelian randomization (TSMR) analysis was performed to examine the causal relationship of UA on MI, and inverse variance-weighted (IVW) results were used as the primary outcome in this study. Sensitivity analysis and horizontal multiple validity test were also performed to verify the reliability of the results.

**Results:**

After multivariable adjustment, individuals with the severe elevation of UA levels have a significantly increased risk of MI (OR=2.843, 95%CI: 1.296-6.237, *P*=0.010). In TSMR analysis, the IVW method demonstrated a significant association between UA and increased risk of MI (OR=1.333, 95%CI: 1.079-1.647, *P*=0.008). Results from the MR-Egger intercept test, Cochran’s Q test, and MR-PRESSO test all suggest the reliability of the IVW analysis. Reverse TSMR analysis did not indicate a causal relationship between genetic susceptibility to MI and UA levels (IVW: OR=1.001, 95%CI: 0.989-1.012, *P*=0.922).

**Conclusion:**

Based on cross-sectional study and Mendelian randomization analysis, it has been demonstrated that UA is an independent risk factor for MI. Elevated levels of UA increase the risk of MI, particularly in cases of severe elevation.

## 1 Introduction

Myocardial infarction (MI) is a severe coronary artery disease that occurs when the formation of plaques on the inner walls of arteries reduce blood flow to the heart, leading to prolonged ischemia and hypoxia in cells, causing cell death and life-threatening conditions (1). It remains a significant cause of death worldwide (2, 3). With the influence of population aging and the coexistence of multiple diseases, the occurrence rate, mortality rate, and case-fatality rate of MI remain high in the elderly (4). Comorbidities affect the prognosis of MI in the elderly. It has been reported that in elderly individuals with a history of MI, especially those with comorbidities such as diabetes and hypertension, the recurrence rate of MI is significantly higher (5). MI imposes a significant economic burden, and increasing awareness of disease-related risk factors and early symptoms can help alleviate the burden of the disease.

Elevated levels of uric acid (UA) are believed to potentially influence the occurrence and prognosis of MI by inducing myocardial cell injury and exacerbating myocardial ischemia-reperfusion injury, making it a potential factor in the progression of MI (6, 7). UA is the final product of human purine metabolism and has a dual role in cardiovascular disease. It acts as an endogenous antioxidant, removing reactive oxygen species and protecting cells from oxidative stress damage, while also promoting cellular oxidative activity (8). Many studies have suggested that elevated UA is associated with MI. For example, a large cross-sectional study by scholars such as Mazidi (9) found that UA was an independent risk factor for MI after correcting for body mass index, hypertension, and type 2 diabetes. However, it has been argued that UA does not increase the risk assessment of coronary heart disease in addition to the traditional risk factors for it (10). In addition to this, a study by Moriarity and other scholars did not find UA to be an independent risk factor for MI (11). Another investigation into the connection between UA and coronary artery disease reported that neither the severity of the condition nor the presence of UA in patients who were male or female (12), but another cross-sectional study later concluded (13) that high UA levels were associated with the severity of coronary artery disease in females.

The controversial findings of these studies may be due to inadequate sample sizes or incomplete adjustment for covariates. The National Health and Nutrition Examination Survey (NHANES) database is a representative nutrition and health status survey program in the United States, which can provide very large sample sizes for cross-sectional study. Therefore, in order to obtain an adequate sample size, in this study we first conducted analyses based on the NHANES database to identify observationally studied associations between UA and MI risk. However, a standalone cross-sectional study alone cannot fully exclude the influence of these confounding factors and reverse causality. Therefore, we used a bidirectional two-sample Mendelian randomization (TSMR) approach to address these issues. TSMR is an analytical method that uses genetic instrumental variables (IVs), specifically single nucleotide polymorphisms (SNPs) that are robustly associated with the exposure factor, to assess causal relationships between exposure and outcomes (14). As IVs are independent of other traits and randomly inherited, bidirectional TSMR analysis can effectively mitigate biases caused by confounding factors and reverse causality often seen in traditional epidemiological research. This approach is increasingly used to evaluate and screen potential causal relationships. Bidirectional TSMR analysis was therefore used in this investigation to evaluate the causative link between UA levels and MI risk.

## 2 Materials and methods

### 2.1 Cross-sectional study

#### 2.1.1 Objects of study

All data were obtained from the NHANES (https://www.cdc.gov/nchs/nhanes/index.htm) database (15). Demographic characteristics of the participants, including age, gender, race, marital status, and education level, as well as lifestyle factors such as smoking status, and health conditions (hypertension, diabetes, hypercholesterolemia and MI), were collected from the population demographics and questionnaire survey data in the database. Data on body mass index (BMI) and UA were obtained from physical measurements and laboratory data.

**Figure 1:**
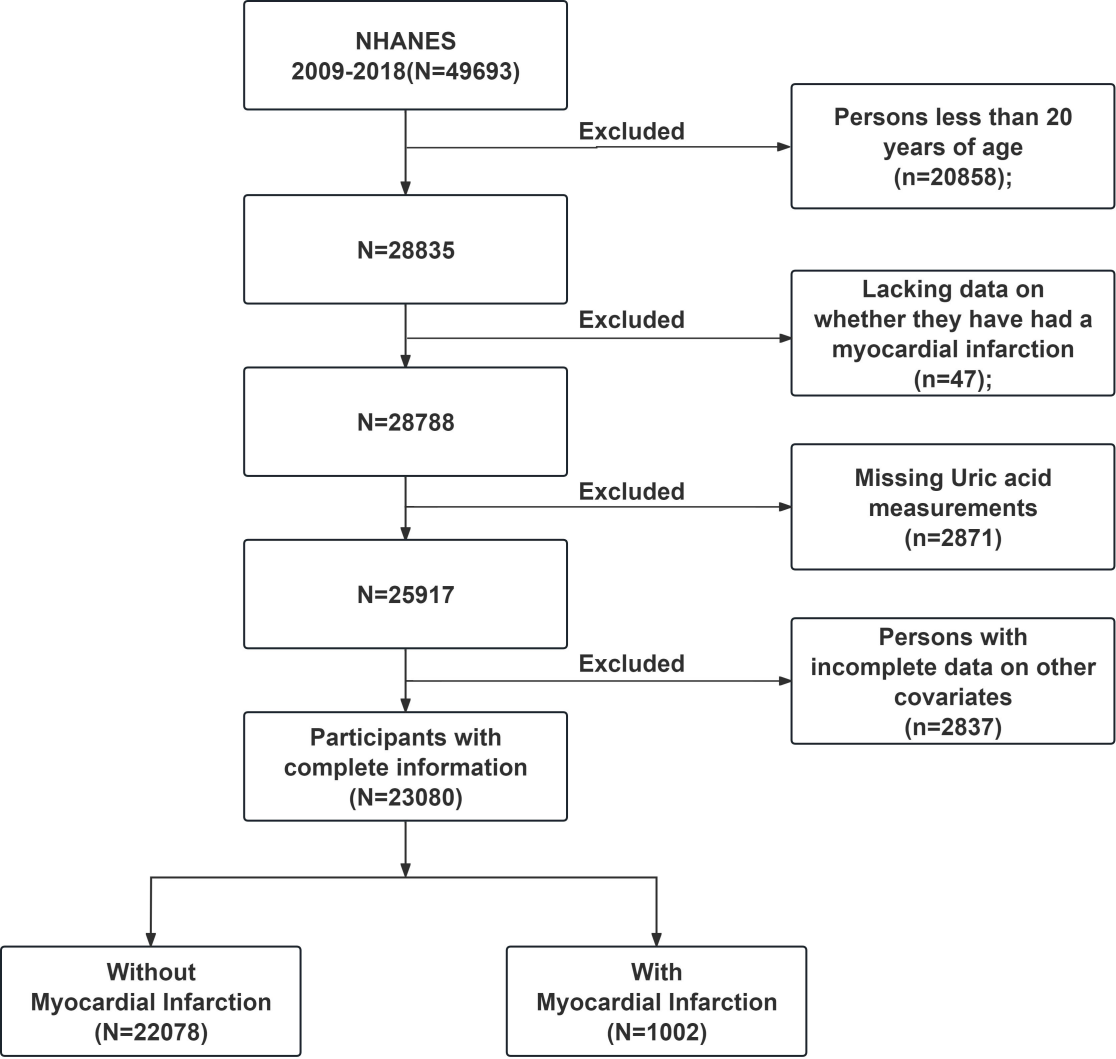
NHANES (2009-2018) data screening flowchart. NHANES, National Health and Nutrition Examination Survey.

We examined data from the past ten years (2009–2018) and strictly adhered to the inclusion and exclusion criteria. The following participants were excluded: (1) individuals under the age of 20, (2) individuals lacking data on whether they had experienced MI, (3) individuals with missing UA measurements, (4) individuals with incomplete data on BMI, race, marital status, education level, smoking status, diabetes, hypertension, or hypercholesterolemia. The final study population consisted of 23,080 individuals, as shown in **Figure 1**. All study participants gave written informed permission, and the National Center for Health Statistics Research Ethics Review Committee in the United States authorized the study.

#### 2.1.2 Sources of UA data

UA data for this study were obtained from NHANES laboratory data. UA measurements were obtained from different laboratory instruments over multiple NHANES cycles. UA measurements were obtained using the Beckman UniCel DxC800 Synchron analyzer in the NHANES 2008-2016 cycles, and the Roche Cobas 6000 analyzer was used for UA measurements in the NHANES 2017-2018 cycles. Combining UA data from multiple NHANES data cycles for analysis has been reported (16). The diagnostic cut-off points for elevated UA levels are defined as UA levels exceeding 420 umol/L for males or 360 umol/L for females (17). Based on UA levels, the participants were divided into four groups: normal group, mild elevation group, moderate elevation group, and severe elevation group. The normal group was defined as UA levels below 420 umol/L for males or 360 umol/L for females. The mild elevation group was defined as UA levels between 420-550 umol/L for males or 360-470 umol/L for females. The moderate elevation group was defined as UA levels exceeding 550 umol/L but below 700 umol/L for males or exceeding 470 umol/L but below 600 umol/L for females. The severe elevation group was defined as UA levels exceeding 700 umol/L for males or exceeding 600 umol/L for females.

#### 2.1.3 MI data collection

Diagnostic data on MI were obtained from the “Medical Condition” questionnaire file of NHANES. Participants were asked if their doctor had ever told them that they had a MI (yes, no, refused to answer, don’t know, and missing data). Subjects who refused to answer, didn’t know, or had missing data were excluded, and the remaining individuals with definite responses proceeded to the next step of screening.

#### 2.1.4 Other covariates

In the study, BMI was calculated by researchers based on measurements of the participants’ height and weight. Smokers included in the study were defined as individuals who had smoked at least 100 cigarettes in their lifetime. The racial categories included Mexican American, Non-Hispanic White, Non-Hispanic Black, and other races (including other Hispanic and other races, such as Multi-Racial). Marital status was categorized as “yes” for individuals who were married and “no” for those who were marked as divorced, separated, living with a partner, widowed, or never married in the demographic characteristics. Education level was categorized into low grade for individuals with “less than 9th grade” and “9-11th grade (Including 12th grade with no diploma)”, and high grade for “high school grad/GED or equivalent”. Individuals with “some college or AA degree”, as well as those with “college graduate or above”, were recategorized as having a college degree or higher. Furthermore, based on relevant studies (18–20), we also incorporated data on hypertension, diabetes, and hyperlipidemia, which were reported by participants in the NHANES database as having been diagnosed with these conditions by a doctor or other health professional during the survey.

#### 2.1.5 Statistical analysis

The statistical analysis in this study was performed using R version 4.3.2. Data from 5 cycles from 2009-2018 were combined and all data were weighted according to the sample weights provided by NHANES for the next analysis. Comparison of general baseline information on whether patients had MI or not was analyzed first. Then, UA was divided into four groups as described in the Methods, A multivariate regression analysis was used to investigate the influence of UA on MI. Finally, The models of logistic regression were created to look into the connections between UA and MI. The crude model was without adjustment for relevant covariates. Age, gender, and racial adjustments were made to Model 1. Model 2 was model 1 adjusted for BMI, marital status, education, and smoking status. Model 3 was adjusted for hypertension, diabetes, and hypercholesterolemia on the basis of model 2. A two-sided *P*<0.05 was considered statistically significant.

### 2.2 MR studies

#### 2.2.1 Research design

**Figure 2:**
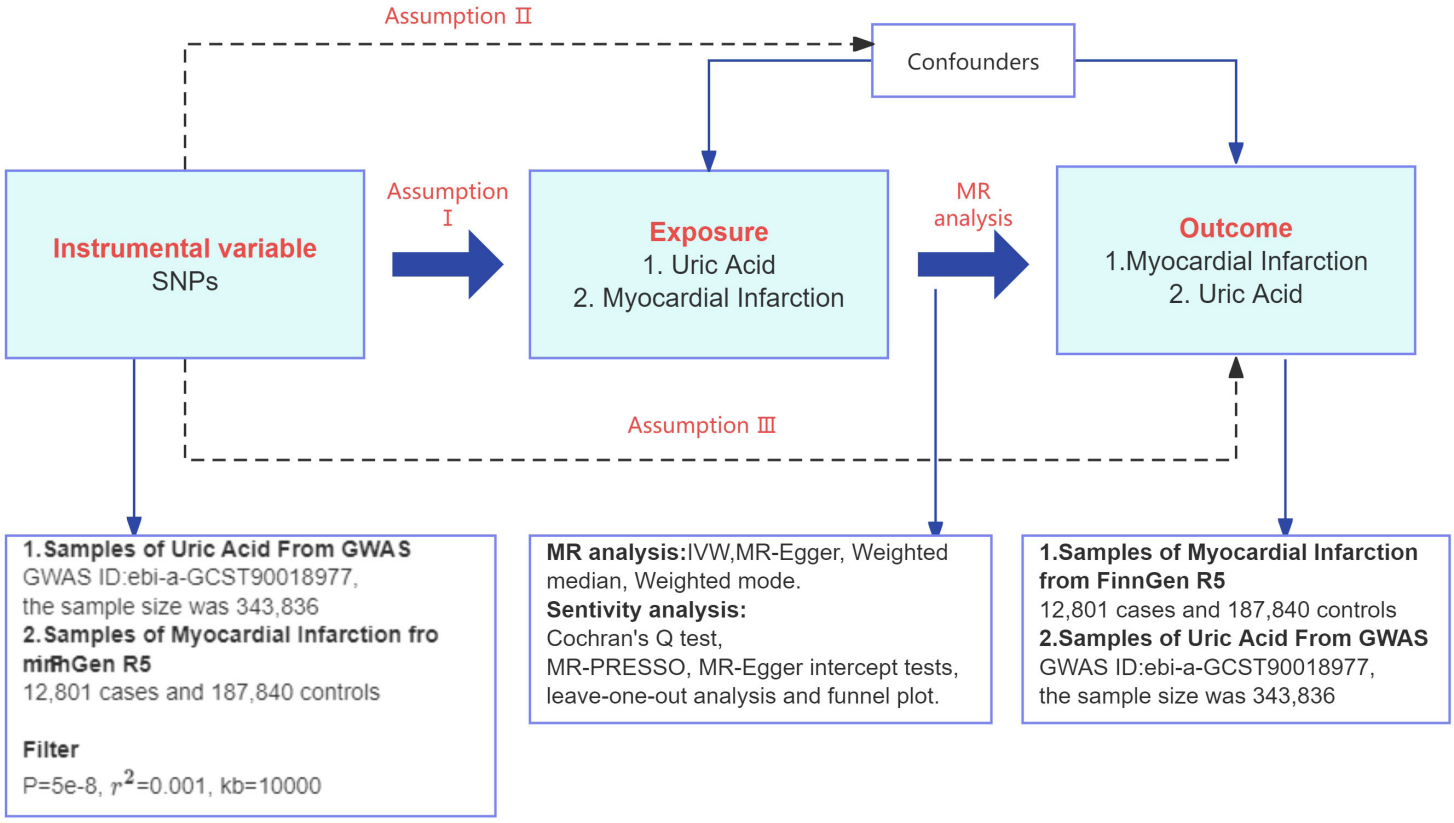
Flowchart of the TSMR study design. GWAS, genome-wide association studies; r^2^, linkage disequilibrium parameter; IVW, inverse variance weighted; MR, Mendelian randomization; SNPs, single nucleotide polymorphisms.

The study design is shown in **Figure 2**. Based on the three assumptions of MR: relevance (assumption I), independence (assumption II), and exclusivity (assumption III), instrumental variable SNP was selected. This study utilized a two-sample MR design, where the exposure was UA, and the outcome was MI.

#### 2.2.2 Source of data

The study employed a two-sample MR study to evaluate the causal relationship between MI risk and UA. All data were sourced from the current publicly accessible Genome-wide association study (GWAS, http://gwas.mrcieu.ac.uk) database. Since ethical informed consent and approvals were done in the original study, no further ethical approval or consent was needed.

Data for UA were obtained from the 2021 Sakaue S open access article (21), which conducted a GWAS of 220 human traits. This large-scale GWAS involved three biobanks: the Biobank Japan, the UK Biobank, and the Finnish Genetic Database, each with specific population backgrounds, and the consistent results across the three biobanks mitigated concerns about the impact of potential bias on the results. The GWAS ID included in this study is ebi-a-GCST90018977, with a sample size of 343,836 individuals, primarily of European ancestry, and SNPs of 19,041,286.

FinnGen, as a biospecimen repository containing both population-based and hospital-based cohorts, enriches a number of disease endpoints that are underrepresented in single cohort-based studies. Thus, for MI GWAS data we selected the finn-b-I9_MI dataset (22), including 12,801 MI patients and 187,840 controls with 16,380,433 SNPs. Individuals with MI were identified in this study according to the International Classification of Diseases standard ICD-10 code I21 diagnosis. MI was defined as acute or with a defined post-onset duration of 4 weeks (28 days) or less.

#### 2.2.3 Instrumental variables selection

As shown in **Figure 2**, MR analyses usually require 3 key assumptions (23): assumption I is that the instrumental variable is selected to be strongly associated with exposure. Assumption II is that there are no potential confounders between exposure and outcome that may be related to the instrumental variable, that is to say, independent. And assumption III is that the instrumental variable affects the outcome only through association with exposure. In order to select valid instrumental variables that take into account the effect of cascading disequilibrium among SNPs, this study screened for SNPs that were independent of each other and had genome-wide significance in terms of the strength of their associations with UA from pooled data from the GWAS of UA, and we followed a rigorous selection procedure from a previous MR study (24). We therefore screened SNPs with *P*< 5×10^-8^, a genetic distance of 10,000 kb, and a threshold of 0.001 for the linkage disequilibrium parameter (r^2^) from the UA data to ensure SNP independence and association. The MI instrumental variable screening for reverse TSMR is the same as the UA instrumental variable screening requirements for forward TSMR.

In order to reduce the weak instrumental variable bias, R^2^ value estimation was performed, and then the F statistic was calculated for each SNP individually, and the weak instrumental variables were removed based on the F test value (25). The formula for the F statistic (26): F=[R^2^×(N-1-K)]/[K×(1-R^2^)]. The variables N, K, and R^2^ denote the number of samples included in the GWAS investigation, the number of SNPs, and the variation of the exposure explained by each instrumental variable separately. R^2^ is calculated as R^2^ = 2×EAF×(1-EAF)×β^2^, where EAF is the allele frequency of the mutation and beta is the effect size of the allele. Instrumental variables with an F-test value less than 30 were removed. Genetic instrumental variable locus-related confounders were removed using the PhenoScanner database (27)(http://www.phenoscanner.medschl.cam.ac.uk/).

#### 2.2.4 Statistical analysis

In this study, the results of inverse-variance weighted (IVW) method (28) were used as the primary outcome, and selected SNPs were used as instrumental variables to assess the causal association between UA and MI. The results of IVW were validated using the results of the MR-Egger method (29), weighted median (30) and weighted mode (31) methods. In addition, the MR Pleiotropy RESidual Sum and Outlier (MR-PRESSO) (32) was used to detect outliers, and if outlying SNPs existed, they were excluded and reanalyzed, and the MR-Egger intercept was used to detect horizontal polytropy, and horizontal pleiotropy existed at *P*< 0.05. Heterogeneity among instrumental variable SNPs was tested using Cochran’s Q test (33), when the Cochran Q test value of Q-Q was *P*> 0.05, it indicated the absence of heterogeneity, suggesting that there was no potential for horizontal polytropy to be examined and provided strong support for the IVW model. Sensitivity testing using Leave-one-out was used to assess outcome stability and to test whether single removal of variance affected the relationship between exposure and outcome.

R software version 4.3.2 was used for all analyses, and the TSMR package had been utilized. Statistical significance was defined as *P*<0.05. The causal associations and sensitivity analysis results have been visualized using forest plots. Scatter plots were used to visualize the effect estimates of each genetic instrumental variable on the exposure and outcome, while funnel plots were used to assess bias.

## 3 Result

### 3.1 NHANES cross-section study

#### 3.1.1 Characterization of subjects’ baseline information

Among the 23080 participants included in this study, 1002 had MI, accounting for 4.5% of the total number. The results of sample weighting indicated that the MI group’s UA was considerably greater than the non-MI group’s (*P*<0.001). **Table 1** displays the variations between the two groups.

**Table 1:** Weighted characteristics of overall participants based on MI grouping, National Health and Nutrition Examination Survey 2009-2018.

Variable	MI		*P* value
	No(22078)	Yes(1002)	
**UA(umol/L), Median(IQR)**	319.910(0.930)	348.756(4.058)	**< 0.001**
**Age(year), Median(IQR)**	47.421(0.266)	64.877(0.449)	**< 0.001**
BMI(kg/m^2^), n(%)			< 0.001
<25	6285(28.73)	218(19.61)	
25-29.9	7212(32.83)	319(31.76)	
>=30	8581(38.44)	465(48.62)	
Elevated UA Subgroup, n(%)			< 0.001
Normal	18109(82.904)	721(74.154)	
Slightly elevated	3543(15.595)	223(21.420)	
Moderately elevated	392(1.405)	52(3.862)	
Severely elevated	34(0.097)	6(0.564)	
Sex, n(%)			< 0.001
female	11652(52.764)	343(36.215)	
male	10426(47.236)	659(63.785)	
Race, n(%)			< 0.001
NH-Black	4678(10.730)	186(9.399)	
NH-White	8703(66.389)	545(74.889)	
Mexican American	3121(8.396)	100(4.322)	
Other races	5576(14.485)	171(11.391)	
Marry, n(%)			0.642
no	10590(43.802)	494(44.788)	
yes	11488(56.198)	508(55.212)	
Education, n(%)			< 0.001
College or above	12406(64.215)	441(50.206)	
High school graduation	4845(21.877)	256(28.668)	
Less than high school	4827(13.909)	305(21.126)	
Hypertension, n(%)			< 0.001
no	14283(68.719)	250(27.796)	
yes	7795(31.281)	752(72.204)	
High Cholesterol, n(%)			< 0.001
no	14556(66.952)	325(29.417)	
yes	7522(33.048)	677(70.583)	
Diabetes, n(%)			< 0.001
no	19156(90.255)	622(64.081)	
yes	2922(9.745)	380(35.919)	
Smoker, n(%)			< 0.001
no	12842(57.531)	358(34.784)	
yes	9236(42.469)	644(65.216)	

UA, uric acid; MI, myocardial infarction; BMI, body mass index; NH-Black, Non-Hispanic Black; NH-White, Non-Hispanic White.

#### 3.1.2 The relationship between UA and MI

**Table 2** shows the correlation between different degrees of elevated UA levels and the risk of developing MI. Weighted logistic regression analysis revealed that UA was positively correlated with MI in models with 4 different moderating variables, and the risk of developing MI was higher in the severely elevated UA group (UA≥700umol/L in men or UA≥600umol/L in women) than in the normal UA group (UA<420umol/L for men or UA<360umol/L for women). In the crude model without controlling for covariates, severely elevated levels of UA were 6.52 times higher than normal UA (OR: 6.522, 95% CI: 3.469-12.262, *P*<0.001). After adjustment for covariates, the results were not significantly affected. The contribution of UA to the risk of MI remained stable in the group with severely elevated UA from model 1 after adjusting for age, sex, and ethnicity to model 3 after fully adjusting for covariates (model 1: OR: 4.226, 95% CI: 2.196-8.133, *P*<0.001, model 2: OR: 3.837, 95% CI. 2.135-6.896, *P*<0.001, model 3: OR: 2.843, 95% CI: 1.296-6.237, *P*=0.010).

**Table 2:** Weighted regression analysis of the causal relationship between UA and MI, National Health and Nutrition Examination Survey 2009-2018.

	Crude mode		Model 1		Model 2		Model 3	
	OR(95%CI)	*P*	OR(95%CI)	*P*	OR(95%CI)	*P*	OR(95%CI)	*P*
**Normal**	ref		ref		ref		ref	
**Slightly elevated**	1.536 (1.207,1.953)	<0.001	1.197 (0.943,1.519)	0.138	1.075 (0.833,1.386)	0.574	0.993 (0.773,1.276)	0.957
**Moderately elevated**	3.074 (2.197,4.300)	<0.001	2.116 (1.480,3.026)	<0.001	1.662 (1.146,2.411)	0.008	1.402 (0.970,2.025)	0.071
**Severely elevated**	6.522 (3.479,12.262)	<0.001	4.226 (2.196,8.133)	<0.001	3.837 (2.135,6.896)	<0.001	2.843 (1.296,6.237)	0.01

UA, uric acid; MI, myocardial infarction; OR, odds ratio; CI, confidence interval. Model 1: adjusted for sex, age, and race. Model 2: adjusted for the variables in model 1 plus BMI, marital status, education, and smoking status. Model 3: adjusted for the variables in model 2 plus hypertension, diabetes, and hypercholesterolemia.

In the crude model, the risk of MI in those with moderately elevated UA (550umol/L≤UA<700umol/L in men or 470umol/L≤UA<600umol/L in women) was 3.07 times higher than that in the group with normal UA (OR: 3.074, 95% CI: 2.197-4.300, *P*<0.001). Moderately elevated UA remained a risk factor for MI after adjusting for age, sex, and race in model 1 (OR: 2.116, 95% CI: 1.480-3.026, *P*<0.001), and after adjusting for the influences of educational attainment, marital status, smoking, and BMI, the OR for model 2 was 1.662 (95% CI: 1.146-2.411, *P*=0.008), but in Model 3, which was fully adjusted for variables, no relationship was found between elevated moderate levels of UA and the occurrence of MI. In addition, we found that at different degrees of elevated UA levels, the OR increased with the degree of elevation.

### 3.2 MR analysis

#### 3.2.1 Results of the selection of instrumental variables

In this study, using UA as an exposure factor, 233 SNPs were extracted as IVs after screening at *P*< 5.0×10^-8^ and excluding the chain imbalance. Strong instrumental variables were selected based on an F-value >30, resulting in the removal of 119 SNPs with F-value below 30. There was no possibility of weak instrumental variable bias. Subsequently, 55 SNPs that were associated with confounding factors were removed. Finally, a total of 59 genome-wide SNPs that were closely related to MI were selected as instrumental variables, and the MR-PRESSO method revealed no outliers. There is no horizontal multiplicity in the MR-Egger intercept test, and the findings are reliable. All detailed SNP information is provided in **Appendix S1–S6**.

#### 3.2.2 Main results of MR analysis

The IVW method showed that UA is a risk factor for MI (OR=1.333, 95% CI: 1.079-1.647). The MR Egger method calculated an OR of 1.616 (95% CI: 1.098-2.378), the Weighted Median method calculated an OR of 1.611 (95% CI: 1.214-2.138), and the Weighted Mode method calculated an OR of 1.759 (95% CI: 1.285-2.409). All P-values were less than 0.05, as shown in **Table 3**.

**Table 3:** Causal link between UA and MI.

method	nsnp	Beta	SE	*P*	OR	95%CI
**Inverse variance weighted**	59	0.288	0.108	0.008	1.333	1.079-1.647
**MR Egger**	59	0.480	0.197	0.018	1.616	1.098-2.378
**Weighted median**	59	0.477	0.144	<0.001	1.611	1.214-2.138
**Weighted mode**	59	0.565	0.160	<0.001	1.759	1.285-2.409

UA, uric acid; MI, myocardial infarction; nsnp, number of single nucleotide polymorphisms, SE, standard error; OR, odds ratio; CI, confidence interval.

**Figure 3:**
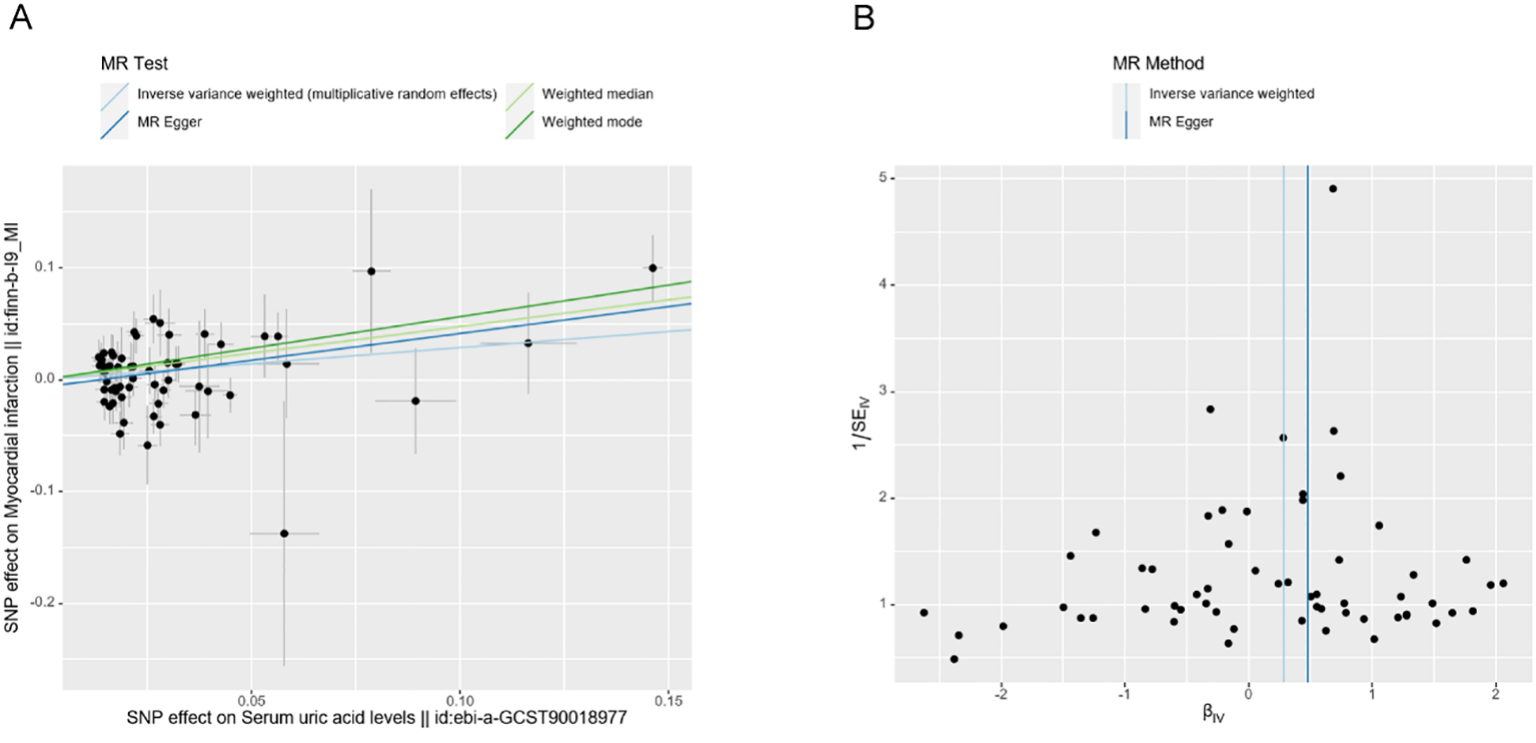
Scatter plots **(A)** and funnel plot **(B)** of the effect of UA on MI.

The scatter plots illustrate the impact of genetically predicted UA levels on the risk of MI occurrence. The direction of β is consistent across the IVW, MR Egger, Weighted Median, and Weighted Mode methods. In this study, the IVW method is considered the primary result (**Figure 3A**). The forest plot presents the effect values of individual SNPs on the outcome (**Appendix D1**).

#### 3.2.3 Evaluation of the robustness and reliability of the study results

Heterogeneity analysis of SNPs showed IVW Q=84.646, *P*=0.013,MR Egger Q=82.681, *P*=0.015.However, the heterogeneity was small with I^2^ = 31.5%, which was negligible by choosing the random effects model of IVW to correct for it in this study. The examination of gene-level pleiotropy was conducted using the MR-Egger regression analysis, and the intercept term was -0.007 (*P*=0.249), with *P*>0.05 indicating that there was no level pleiotropy. **Table 4** presents the findings.

**Table 4:** MR heterogeneity and horizontal multiple validity tests.

IVW Cochran’s Q	IVW Q *P* value	MR Egger Cochran’s Q	MR Egger Q *P* value	I^2^	MR Egger intercept	MR Egger Intercept *P* value
84.646	0.013	82.681	0.015	31.5%	-0.007	0.249

MR, Mendelian randomization; IVW, Inverse variance weighted.

The leave-one-out method did not significantly change the effect of the remaining SNPs on the results after sequentially removing individual SNPs in the leave-one-out test, indicating that there were no outlier SNPs or SNPs that affected the results (**Appendix D2**). Additionally, the funnel plot demonstrates a symmetrical distribution of data points, suggesting a low potential for underlying bias in the results obtained using the 59 SNPs as instrumental variables (**Figure 3B**). This indicates that the results are stable and reliable.

#### 3.2.4 Reverse TSMR analysis

MI was the exposure factor and UA was the outcome variable in reverse TSMR. A total of 11 SNPs were evaluated and found to be IVs, all with F-values>10 (**Appendix S5**). The horizontal pleiotropy test (Egger’s intercept= -0.002, *P*=0.496) (**Table 5**) found no evidence of horizontal pleiotropy. The MR findings did not show a link between genetic vulnerability to MI and elevated UA levels (IVW, OR: 1.001, 95% CI: 0.989-1.012, *P*=0.922). Other techniques’ results were MR-Egger (OR: 1.012, 95% CI: 0.978-1.048, *P*=0.501), weighted median (OR: 0.998, 95% CI: 0.986-1.009, *P*=0.705) and weighted mode (OR: 0.998, 95% CI: 0.985-1.010, *P*=0.737) (**Table 6**; **Appendix D3, D4**). Among the results of the heterogeneity test, MR-Egger regression showed relatively small heterogeneity (Cochran’s Q=17.47, *P*=0.042), and relatively small heterogeneity among IVs was also found for IVW (Cochran's Q=18.449, *P*=0.048) (**Table 5**). The MR-PRESSO showed that the Global Test had no horizontal polytropy (Global Test RSSobs=20.87, *P*=0.073) and no outliers were observed. The “leave-one-out” method and the forest plot are shown in the **Appendix D5, D6**.

**Table 5:** Reverse MR heterogeneity and horizontal multiplicity tests.

IVW Cochran’s Q	IVW Q *P* value	MR Egger Cochran’s Q	MR Egger Q *P* value	I^2^	MR Egger intercept	MR Egger Intercept *P* value
18.449	0.048	17.470	0.042	45.8%	-0.002	0.496

MR, Mendelian randomization; IVW, Inverse variance weighted.

**Table 6:** Reverse MI and UA causal association.

method	nsnp	Beta	SE	*P*	OR	95%CI
**Inverse variance weighted**	11	0.001	0.006	0.922	1.001	0.989-1.012
**MR Egger**	11	0.012	0.017	0.501	1.012	0.978-1.048
**Weighted median**	11	-0.002	0.006	0.705	0.998	0.986-1.009
**Weighted mode**	11	-0.002	0.006	0.737	0.998	0.985-1.010

UA, uric acid; MI, myocardial infarction; nsnp, number of single nucleotide polymorphisms, SE, standard error; OR, odds ratio; CI, confidence interval.

## 4 Discussion

We observed higher UA levels in patients with MI using NHANES data, and only severely elevated UA levels were an independent risk factor for MI after multifactorial regression analysis correcting for influencing factors, and then explored the causal connection between UA levels and risk of MI by two-way two-sample Mendelian randomization. Genetically predicted high blood UA levels were associated with a high risk of developing MI (IVW, OR=1.333, 95% CI:1.079-1.647, *P*=0.008), and inverse MR analysis did not demonstrate a causal relationship between MI on elevated UA levels (IVW, OR: 1.001,95% CI: 0.989-1.012, *P*=0.922). All these results indicated that UA plays an important role in MI. The sensitivity analysis showed that the results of MR analysis were reliable.

Numerous observational studies have examined the connection between UA and MI, and the majority of them have concluded that increased UA poses a risk for MI development. And that the prognosis of MI is impacted by elevated UA levels. For example, a large European prospective study (34) concluded that UA is a strong risk factor for MI, and another study reported (35) that UA remained an independent risk factor for MI after adjusting for possible confounders. In the research conducted by Mehrpooya and colleagues (36), it was observed that higher Killip classifications were associated with increased UA levels in patients with ST-elevation MI, suggesting that UA might be an indicator of worse clinical outcomes in these patients. Furthermore, certain studies indicate that UA can be regarded as a biological marker to distinguish ST-elevation MI from other types of coronary heart disease (37, 38). The results of our cross-sectional study with information extracted from the NHANES database are consistent with these studies, which found that elevated UA levels were positively associated with MI and that severe elevated UA levels were a significant risk factor for the development of MI.

Nevertheless, two clinical studies have shown that pharmacologic lowering of UA levels does not improve cardiovascular outcomes and reduce cardiovascular events in patients with ischemic heart disease (39, 40). On the one hand, this may be because the populations of these two studies were patients who already had cardiovascular disease, and even with pharmacologic UA-lowering therapy, it is difficult to change irreversible changes that may already exist in the patients. On the other hand, and as we have said, the role of UA in cardiovascular disease has been a focus of attention. Our cross-sectional study using data from the NHANES database validated the association between elevated UA levels and MI. However, we did not explore in depth the effect of elevated UA levels on the prognostic outcome of MI, and the scope of our study was more limited compared with these two studies. In the future, we plan to conduct a large-sample randomized controlled trial to further confirm the relationship between UA and adverse outcomes in MI.

The exact mechanism of the association between UA and MI has not been fully clarified, and current studies generally agree (41) that elevated UA has the effect of promoting oxidative stress, inflammation, and endothelial dysfunction in endothelial cells, which makes vascular smooth muscle cells proliferate and vasoconstriction, and exacerbates tissue hypoxia, and is an important pathologic mechanism by which elevated UA affects the occurrence of MI. Among them, inflammation plays a major role in UA-induced cardiomyocyte injury, closely related to the activation of the NOD-like receptor pyrin domain-containing protein 3 (NLRP3) inflammasome. UA was reported to induce ROS production through the activation of NLRP3 inflammasome (42). Subsequently, an experimental animal study demonstrated (6) that myocardial ischemia-reperfusion injury was aggravated through the ROS/NLRP3 inflammasome-mediated pyroptosis pathway. Another *in vivo in vitro* experiment shown further (7) that high concentrations of UA may induce cardiomyocyte injury through activation of NLRP3 and ROS/TRPM2 channels/Ca2+. In addition, through a variety of methods, including blocking L-arginine absorption and stimulating L-arginine breakdown by arginase, UA can result in lower NO production and bioavailability (43). Meanwhile, the inflammatory response to tissue hypoxia during MI induces XO enzymes, which increase XO activity thereby increasing UA levels and ROS, promoting oxidative stress and leading to endothelial dysfunction (44). It has also been found (45) that elevated UA levels induce endothelial dysfunction through mitochondrial calcium overload mediated by mitochondrial Na+/Ca2+ exchangers.

The results of TSMR analysis by Efstathiadou (46) indicated that there was little proof of a clinically relevant causal effect of genetically determined UA on a range of cardiovascular diseases, including MI. In contrast, our results are not consistent with this; the forward MR results of the present study revealed a causal relationship between UA and the risk of MI occurrence and excluded the effect of reverse causality.The difference in conclusions may be attributed to the fact that Efstathiadou studied a wider range of cardiovascular diseases, and that the GWAS dataset for coronary artery disease selected for analysis in his study contains a different number of SNPs as well as a different sample size than the GWAS dataset we selected for MI. Therefore, in the future, more MR methods can be applied, such as the use of mediator MR methods to analyze the effects of different SNP-related mediators on the results, to be able to more comprehensively analyze the relationship between UA and MI, and thus obtain more reliable conclusions.

This study’s mix of observational research with two-way TSMR analysis is its strongest point. First, a cross-sectional research using the NHANES database examined the connection between UA levels and MI risk in our investigation, but questionnaire-based MI data are retrospective diagnostics with the potential for recall bias as well as nonresponse bias. Therefore, the use of two-way TSMR analysis addresses the effects of measurement bias, nonresponse bias, and recall bias in observational studies and avoids the influence of reverse causality on the results, further illustrating the causal relationship between UA levels and MI. Thus, our study is of fundamental importance in that it attempts to strengthen our comprehension of the causal relationship between UA and MI in a genomic context. In addition, there are some limitations of the study. The data on MI were obtained from questionnaires, and the type of MI was not specified, thus further stratified analyses could not be performed to explore the relationship between UA levels and different types of MI. In addition, the sensitivity analyses’ results in the MR analysis were inconsistent, making it unable to fully rule out the effect of possibly confounding SNPs. Finally, the cross-sectional study and MR analysis data were not from the same sample; the study population for the data from NHANES was American, whereas the MR analysis population was participants of European ancestry, which makes our results regionally limited. These need to be investigated in more studies in the future.

## 5 Conclusion

In conclusion, our cross-sectional study supports that UA is an independent risk factor for MI. Despite potential uncontrolled confounding factors that may affect observational results, by incorporating further TSMR analysis, we have discovered a causal link between increased UA levels and MI, increasing the risk of MI. These findings need to be confirmed by additional research, and the underlying mechanisms require further exploration. Future research on the function of UA in the prevention and treatment of MI may use these findings as a reference.

## Data Availability

The original contributions presented in the study are included in the article/**Supplementary Material**. Further inquiries can be directed to the corresponding author.
