# Supplementary material for: Uric acid is associated with increased risk of myocardial infarction: results from NHANES 2009-2018 and bidirectional two-sample Mendelian randomization analysis
Source: Front Endocrinol (Lausanne). 2024 Oct 18;15:1424070. doi: 10.3389/fendo.2024.1424070 (PMC11527614; doi:10.3389/fendo.2024.1424070)
Supplement: Supplementary file 6 [file Presentation1.pptx]

## Slide 1
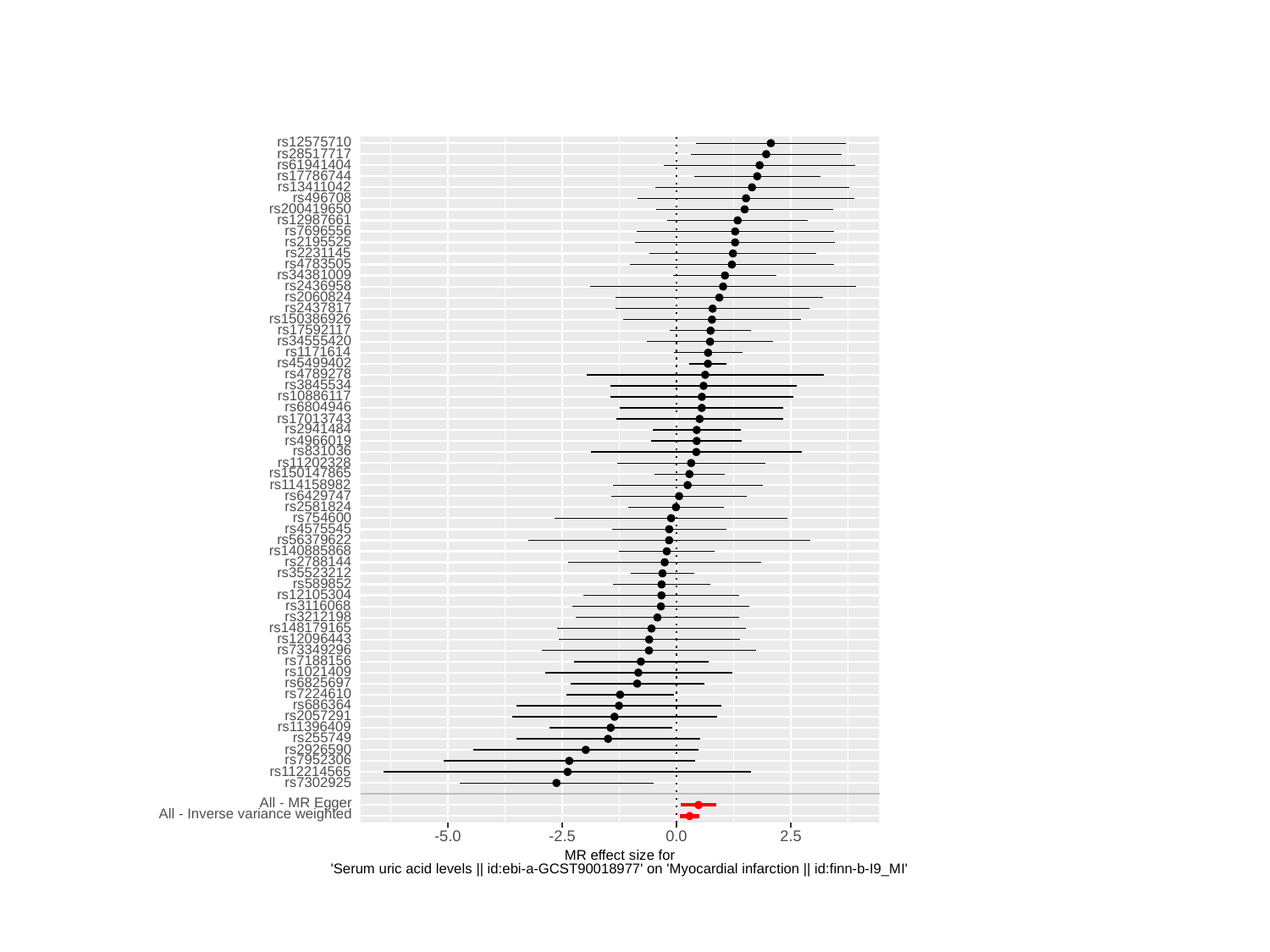

#
rs12575710
rs28517717
rs61941404
rs17786744
rs13411042
rs496708
rs200419650
rs12987661
rs7696556
rs2195525
rs2231145
rs4783505
rs34381009
rs2436958
rs2060824
rs2437817
rs150386926
rs17592117
rs34555420
rs1171614
rs45499402
rs4789278
rs3845534
rs10886117
rs6804946
rs17013743
rs2941484
rs4966019
rs831036
rs11202328
rs150147865
rs114158982
rs6429747
rs2581824
rs754600
rs4575545
rs56379622
rs140885868
rs2788144
rs35523212
rs589852
rs12105304
rs3116068
rs3212198
rs148179165
rs12096443
rs73349296
rs7188156
rs1021409
rs6825697
rs7224610
rs686364
rs2057291
rs11396409
rs255749
rs2926590
rs7952306
rs112214565
rs7302925
All - MR Egger
All - Inverse variance weighted
-5.0
-2.5
0.0
2.5
MR effect size for
'Serum uric acid levels || id:ebi-a-GCST90018977' on 'Myocardial infarction || id:finn-b-I9_MI'
