# Supplementary material for: Uric acid is associated with increased risk of myocardial infarction: results from NHANES 2009-2018 and bidirectional two-sample Mendelian randomization analysis
Source: Front Endocrinol (Lausanne). 2024 Oct 18;15:1424070. doi: 10.3389/fendo.2024.1424070 (PMC11527614; doi:10.3389/fendo.2024.1424070)
Supplement: Supplementary file 7 [file Presentation2.pptx]

## Slide 1
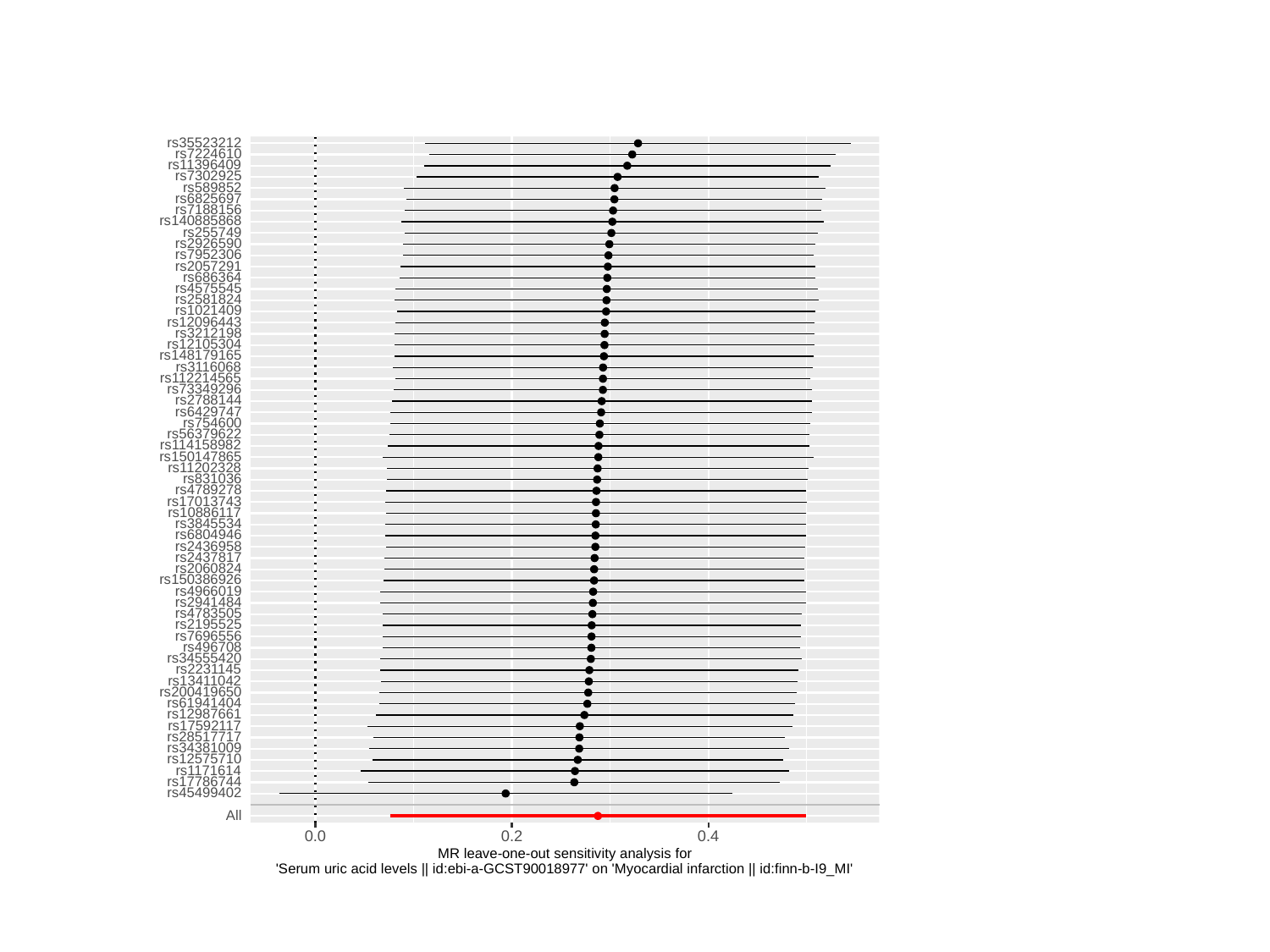

#
rs35523212
rs7224610
rs11396409
rs7302925
rs589852
rs6825697
rs7188156
rs140885868
rs255749
rs2926590
rs7952306
rs2057291
rs686364
rs4575545
rs2581824
rs1021409
rs12096443
rs3212198
rs12105304
rs148179165
rs3116068
rs112214565
rs73349296
rs2788144
rs6429747
rs754600
rs56379622
rs114158982
rs150147865
rs11202328
rs831036
rs4789278
rs17013743
rs10886117
rs3845534
rs6804946
rs2436958
rs2437817
rs2060824
rs150386926
rs4966019
rs2941484
rs4783505
rs2195525
rs7696556
rs496708
rs34555420
rs2231145
rs13411042
rs200419650
rs61941404
rs12987661
rs17592117
rs28517717
rs34381009
rs12575710
rs1171614
rs17786744
rs45499402
All
0.0
0.2
0.4
MR leave-one-out sensitivity analysis for
'Serum uric acid levels || id:ebi-a-GCST90018977' on 'Myocardial infarction || id:finn-b-I9_MI'
