# Supplementary material for: Uric acid is associated with increased risk of myocardial infarction: results from NHANES 2009-2018 and bidirectional two-sample Mendelian randomization analysis
Source: Front Endocrinol (Lausanne). 2024 Oct 18;15:1424070. doi: 10.3389/fendo.2024.1424070 (PMC11527614; doi:10.3389/fendo.2024.1424070)
Supplement: Supplementary file 8 [file Presentation3.pptx]

## Slide 1
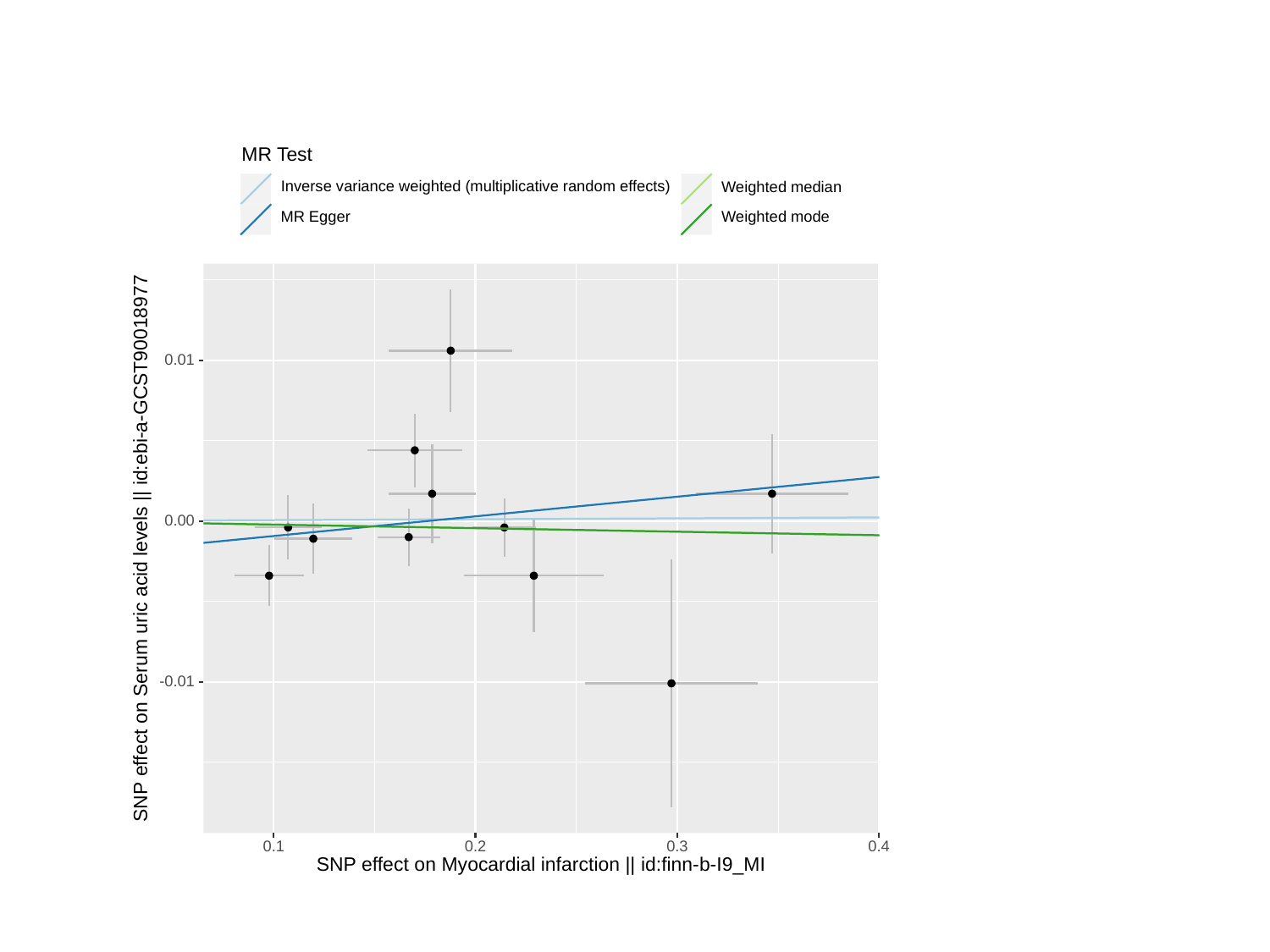

#
MR Test
Inverse variance weighted (multiplicative random effects)
Weighted median
MR Egger
Weighted mode
0.01
0.00
SNP effect on Serum uric acid levels || id:ebi-a-GCST90018977
-0.01
0.3
0.1
0.2
0.4
SNP effect on Myocardial infarction || id:finn-b-I9_MI
