# Supplementary material for: Uric acid is associated with increased risk of myocardial infarction: results from NHANES 2009-2018 and bidirectional two-sample Mendelian randomization analysis
Source: Front Endocrinol (Lausanne). 2024 Oct 18;15:1424070. doi: 10.3389/fendo.2024.1424070 (PMC11527614; doi:10.3389/fendo.2024.1424070)
Supplement: Supplementary file 9 [file Presentation4.pptx]

## Slide 1
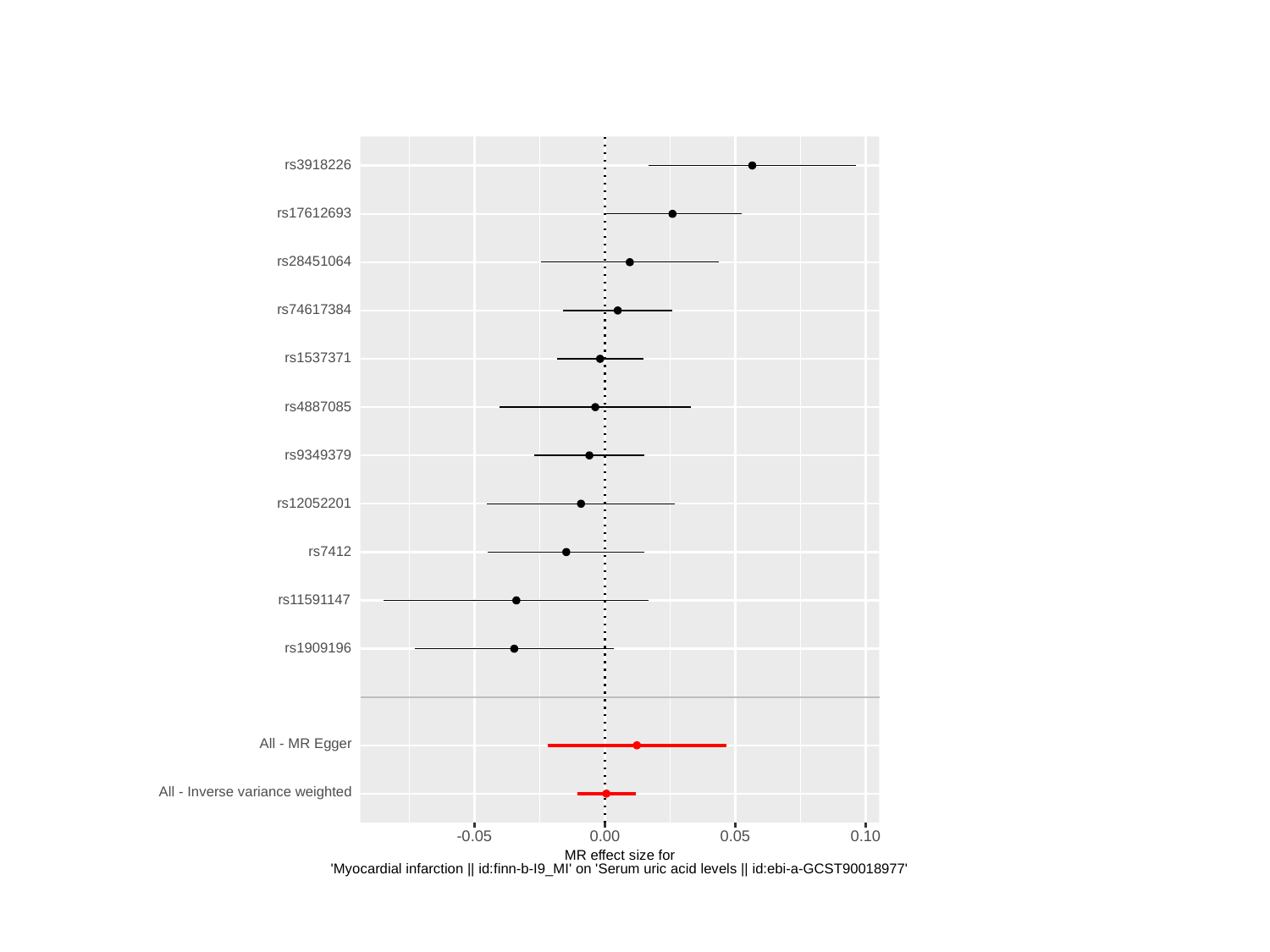

#
rs3918226
rs17612693
rs28451064
rs74617384
rs1537371
rs4887085
rs9349379
rs12052201
rs7412
rs11591147
rs1909196
All - MR Egger
All - Inverse variance weighted
-0.05
0.00
0.05
0.10
MR effect size for
'Myocardial infarction || id:finn-b-I9_MI' on 'Serum uric acid levels || id:ebi-a-GCST90018977'
