# Supplementary material for: Uric acid is associated with increased risk of myocardial infarction: results from NHANES 2009-2018 and bidirectional two-sample Mendelian randomization analysis
Source: Front Endocrinol (Lausanne). 2024 Oct 18;15:1424070. doi: 10.3389/fendo.2024.1424070 (PMC11527614; doi:10.3389/fendo.2024.1424070)
Supplement: Supplementary file 10 [file Presentation5.pptx]

## Slide 1
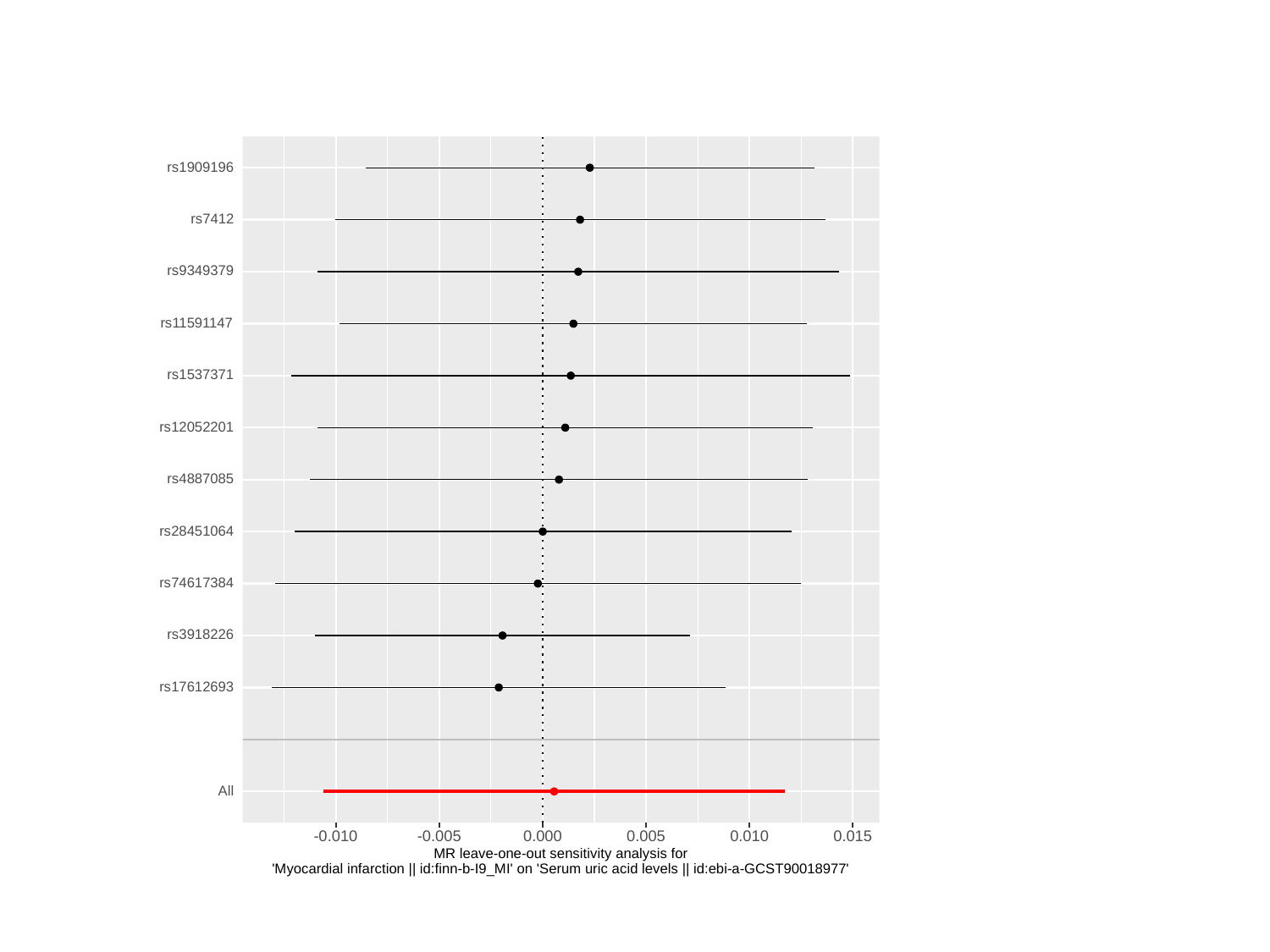

#
rs1909196
rs7412
rs9349379
rs11591147
rs1537371
rs12052201
rs4887085
rs28451064
rs74617384
rs3918226
rs17612693
All
-0.010
-0.005
0.000
0.005
0.010
0.015
MR leave-one-out sensitivity analysis for
'Myocardial infarction || id:finn-b-I9_MI' on 'Serum uric acid levels || id:ebi-a-GCST90018977'
