# Supplementary figures and images for: Uric acid is associated with increased risk of myocardial infarction: results from NHANES 2009-2018 and bidirectional two-sample Mendelian randomization analysis
Source: Front Endocrinol (Lausanne). 2024 Oct 18;15:1424070. doi: 10.3389/fendo.2024.1424070 (PMC11527614; doi:10.3389/fendo.2024.1424070)

## Slide 1
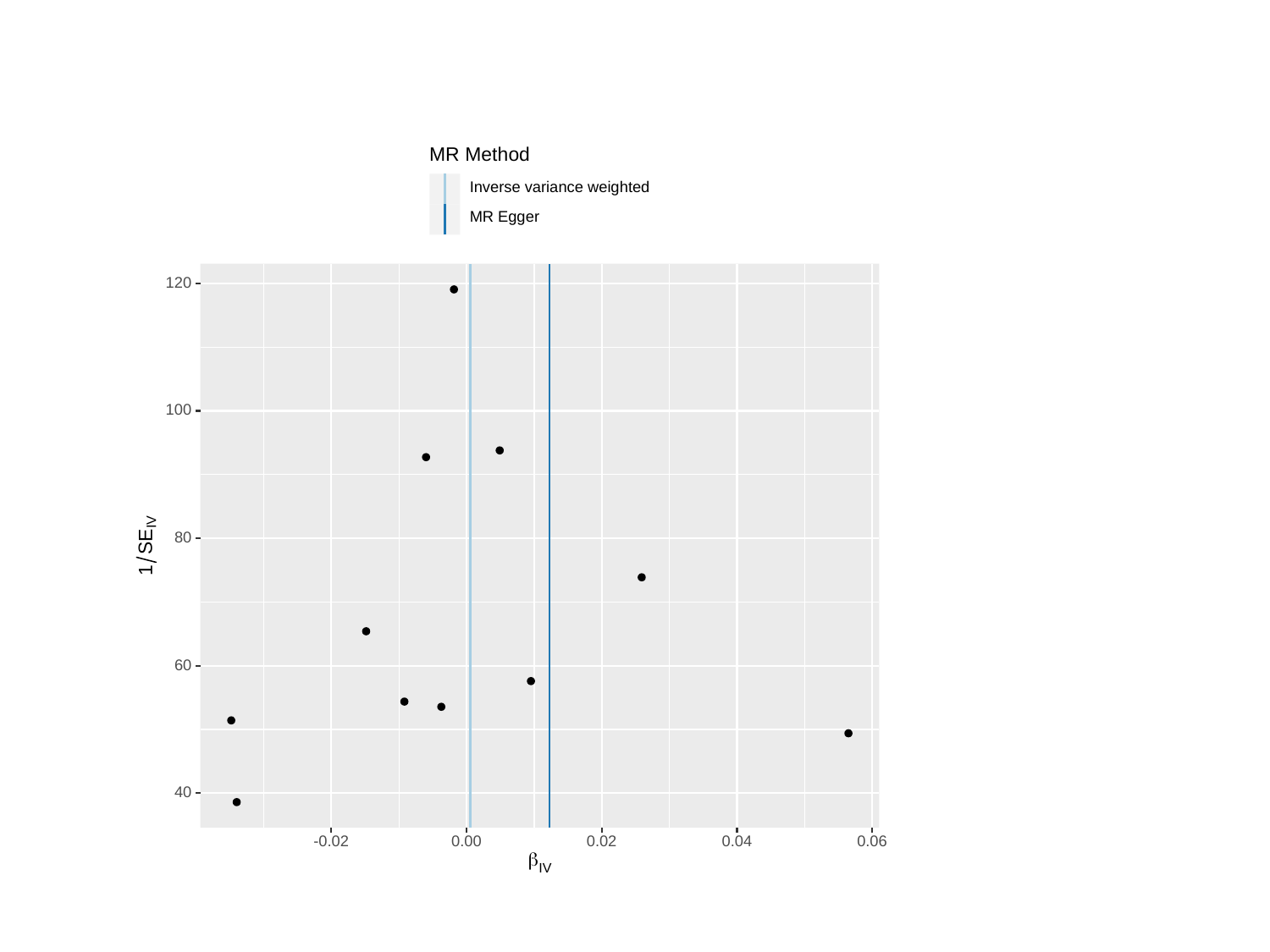

#
MR Method
Inverse variance weighted
MR Egger
120
100
V
I
E
80
S
1
60
40
-0.02
0.00
0.02
0.04
0.06
β
I
V

Supplement: Supplementary file 11 [file Presentation6.pptx]
