# Supplementary material for: Uric acid is associated with increased risk of myocardial infarction: results from NHANES 2009-2018 and bidirectional two-sample Mendelian randomization analysis
Source: Front Endocrinol (Lausanne). 2024 Oct 18;15:1424070. doi: 10.3389/fendo.2024.1424070 (PMC11527614; doi:10.3389/fendo.2024.1424070)
Supplement: Supplementary file 12 [file Table1.docx]

**STROBE-MR checklist of recommended items to address in reports of Mendelian randomization studies**^1^ ^2^

| **Item No.** | **Section** | **Checklist item** | **Relevant text from manuscript** |
| --- | --- | --- | --- |
| 1 | **TITLE and ABSTRACT** | Indicate Mendelian randomization (MR) as the study’s design in the title and/or the abstract if that is a main purpose of the study | Uric acid is associated with increased risk of myocardial infarction: Results from NHANES 2009-2018 and bidirectional two-sample Mendelian randomization analysis.  Abstract  Aims  Although a growing number of studies have shown that elevated uric acid (UA) levels are associated with multiple cardiovascular risk factors and progression of coronary artery disease. However, the causal relationship between UA and the occurrence of myocardial infarction (MI) remains uncertain. The aim of this study was to investigate the relationship between UA and the risk of MI.  Methods  We screened 23,080 patients in the National Health and Nutrition Examination Survey (NHANES) database for the decade 2009-2018 and explored the association between UA and MI risk using multivariate logistic regression modeling. In addition, a two-way two-sample Mendelian randomization (TSMR) analysis was performed to examine the causal relationship of UA on MI, and IVW results were used as the primary outcome in this study. Sensitivity analysis and horizontal multiple validity test were also performed to verify the reliability of the results.  Results  According to observational studies, there is a positive correlation between elevated levels of UA and MI. After multivariable adjustment, individuals with severe elevation of UA levels have a significantly increased risk of MI (OR=2.843, 95%CI: 1.296-6.237, P=0.010). In TSMR analysis, the IVW method demonstrated a significant association between UA and increased risk of MI (OR=1.333, 95%CI: 1.079-1.647, P=0.008). Results from MR-Egger intercept test, Cochran's Q test, and MR-PRESSO test all suggest the reliability of the IVW analysis. Reverse TSMR analysis did not indicate a causal relationship between genetic susceptibility to MI and UA levels (IVW: OR=1.001, 95%CI: 0.989-1.012, P=0.922).  Conclusion  Based on cross-sectional studies and Mendelian randomization analysis, it has been demonstrated that UA is an independent risk factor for MI. Elevated levels of UA increase the risk of MI, particularly in cases of severe elevation.  Keywords: uric acid, myocardial infarction, NHANES, cross-sectional study, Mendelian randomization |
|  | **INTRODUCTION** |  |  |
| 2 | **Background** | Explain the scientific background and rationale for the reported study. What is the exposure? Is a potential causal relationship between exposure and outcome plausible? Justify why MR is a helpful method to address the study question | Myocardial infarction (MI) is a severe coronary artery disease that occurs when the formation of plaques on the inner walls of arteries reduces blood flow to the heart, leading to prolonged ischemia and hypoxia in cells, causing cell death and life-threatening conditions. It remains a significant cause of death worldwide. With the influence of population aging and the coexistence of multiple diseases, the occurrence rate, mortality rate, and case-fatality rate of MI remain high in the elderly. Comorbidities affect the prognosis of MI in the elderly. It has been reported that in elderly individuals with a history of MI, especially those with comorbidities such as diabetes and hypertension, the recurrence rate of MI is significantly higher. MI imposes a significant economic burden, and increasing awareness of disease-related risk factors and early symptoms can help alleviate the burden of the disease.  Elevated levels of uric acid (UA) are believed to potentially influence the occurrence and prognosis of MI through inducing myocardial cell injury and exacerbating myocardial ischemia-reperfusion injury, making it a potential factor in the progression of MI. UA is the final product of human purine metabolism and has a dual role in cardiovascular disease. It acts as an endogenous antioxidant, removing reactive oxygen species and protecting cells from oxidative stress damage, while also promoting cellular oxidative activity. Many studies have suggested that elevated UA is associated with MI. For example, a large cross-sectional study by scholars such as Mazidi found that UA was an independent risk factor for MI after correcting for body mass index, hypertension, and type 2 diabetes. However, it has been argued that UA does not increase the risk assessment of coronary heart disease in addition to the traditional risk factors forit. In addition to this, a study by Moriarity and other scholars did not find UA to be an independent risk factor for MI. Another investigation into the connection between UA and coronary artery disease reported that neither the severity of the condition nor the presence of UA in patients who were male or female, but another cross-sectional study later concluded that high UA levels were associated with the severity of coronary artery disease in females.  The controversial findings of these studies may be due to inadequate sample sizes or incomplete adjustment for covariates. The National Health and Nutrition Examination Survey (NHANES) database is a representative nutrition and health status survey program in the United States, which can provide very large sample sizes for cross-sectional studies. Therefore, in order to obtain an adequate sample size, in this study we first conducted analyses based on the NHANES database to identify observationally studied associations between UA and MI risk. However, a standalone cross-sectional study alone cannot fully exclude the influence of these confounding factors and reverse causality. Therefore, we used a bidirectional two-sample Mendelian randomization (TSMR) approach to address these issues. This study utilized a two-sample MR design, where the exposure was UA, and the outcome was MI. |
| 3 | **Objectives** | State specific objectives clearly, including pre-specified causal hypotheses (if any). State that MR is a method that, under specific assumptions, intends to estimate causal effects | TSMR is an analytical method that uses genetic instrumental variables (IVs), specifically single nucleotide polymorphisms (SNPs) that are robustly associated with the exposure factor, to assess causal relationships between exposure and outcomes. As IVs are independent of other traits and randomly inherited, bidirectional TSMR analysis can effectively mitigate biases caused by confounding factors and reverse causality often seen in traditional epidemiological research. This approach is increasingly used to evaluate and screen potential causal relationships. Bidirectional TSMR analysis was therefore used in this investigation to evaluate the causative link between UA levels and MI risk. |
|  | **METHODS** |  |  |
| 4 | **Study design and data sources** | Present key elements of the study design early in the article. Consider including a table listing sources of data for all phases of the study. For each data source contributing to the analysis, describe the following: |  |
|  | a) | Setting: Describe the study design and the underlying population, if possible. Describe the setting, locations, and relevant dates, including periods of recruitment, exposure, follow-up, and data collection, when available. | The study employed a two-sample MR study to evaluate the causal relationship between MI risk and UA. All data were sourced from the current publicly accessible Genome-wide association study (GWAS, http://gwas.mrcieu.ac.uk) database. |
|  | b) | Participants: Give the eligibility criteria, and the sources and methods of selection of participants. Report the sample size, and whether any power or sample size calculations were carried out prior to the main analysis | Data for UA were obtained from the 2021 Sakaue S open access article, which conducted a GWAS of 220 human traits. This large-scale GWAS involved three biobanks: the Biobank Japan , the UK Biobank, and the Finnish Genetic Database, each with specific population backgrounds, and the consistent results across the three biobanks mitigated concerns about the impact of potential bias on the results. The GWAS ID included in this study is ebi-a-GCST90018977, with a sample size of 343,836 individuals, primarily of European ancestry, and SNPs of 19,041,286.  FinnGen, as a biospecimen repository containing both population-based and hospital-based cohorts, enriches a number of disease endpoints that are underrepresented in single cohort-based studies. Thus, for MI GWAS data we selected the finn-b-I9_MI dataset, including 12,801 MI patients and 187,840 controls with 16,380,433 SNPs. Individuals with MI were identified in this study according to the International Classification of Diseases standard ICD-10 code I21 diagnosis.MI was defined as acute or with a defined post-onset duration of 4 weeks (28 days) or less. |
|  | c) | Describe measurement, quality control and selection of genetic variants | In order to select valid instrumental variables that take into account the effect of cascading disequilibrium among SNPs, this study screened for SNPs that were independent of each other and had genome-wide significance in terms of the strength of their associations with UA from pooled data from the GWAS of UA, and we followed a rigorous selection procedure from a previous MR study. |
|  | d) | For each exposure, outcome, and other relevant variables, describe methods of assessment and diagnostic criteria for diseases | We therefore screened SNPs with P< 5×10-8, a genetic distance of 10,000 kb, and a threshold of 0.001 for the linkage disequilibrium parameter (r^2^) from the UA data to ensure SNP independence and association. The MI instrumental variable screening for reverse TSMR is the same as the UA instrumental variable screening requirements for forward TSMR. |
|  | e) | Provide details of ethics committee approval and participant informed consent, if relevant | Since ethical informed consent and approvals were done in the original study, no further ethical approval or consent was needed. |
| 5 | **Assumptions** | Explicitly state the three core IV assumptions for the main analysis (relevance, independence and exclusion restriction) as well assumptions for any additional or sensitivity analysis | Based on the three assumptions of MR: relevance (assumption I), independence (assumption II), and exclusivity (assumption III), instrumental variable SNP was selected. MR analyses usually require 3 key assumptions: assumption I is that the instrumental variable is selected to be strongly associated with exposure. Assumption II is that there are no potential confounders between exposure and outcome that may be related to the instrumental variable, that is to say, independent. And assumption III is that the instrumental variable affects the outcome only through association with exposure. |
| 6 | **Statistical methods: main analysis** | Describe statistical methods and statistics used |  |
|  | a) | Describe how quantitative variables were handled in the analyses (i.e., scale, units, model) | In order to reduce the weak instrumental variable bias, R^2^ value estimation was performed, and then the F statistic was calculated for each SNP individually, and the weak instrumental variables were removed based on the F test value. |
|  | b) | Describe how genetic variants were handled in the analyses and, if applicable, how their weights were selected | Genetic instrumental variable locus-related confounders were removed using the PhenoScanner database(http://www.phenoscanner.medschl.cam.ac.uk/). |
|  | c) | Describe the MR estimator (e.g. two-stage least squares, Wald ratio) and related statistics. Detail the included covariates and, in case of two-sample MR, whether the same covariate set was used for adjustment in the two samples | The formula for the F statistic: F=[R^2^×(N-1-K)]/[K×(1-R^2^)]. The variables N, K, and R^2^ denote the number of samples included in the GWAS investigation, the number of SNPs, and the variation of the exposure explained by each instrumental variable separately. R^2^ is calculated as R^2^ = 2×EAF×(1-EAF)×β^2^, where EAF is the allele frequency of the mutation and beta is the effect size of the allele. Instrumental variables with an F-test value less than 30 were removed. |
|  | d) | Explain how missing data were addressed | None. |
|  | e) | If applicable, indicate how multiple testing was addressed | Inapplicable. |
| 7 | **Assessment of assumptions** | Describe any methods or prior knowledge used to assess the assumptions or justify their validity | In this study, the results of inverse-variance weighted (IVW) method were used as the primary outcome, and selected SNPs were used as instrumental variables to assess the causal association between UA and MI. The results of IVW were validated using the results of the MR-Egger method, weighted median and weighted mode methods. |
| 8 | **Sensitivity analyses and additional analyses** | Describe any sensitivity analyses or additional analyses performed (e.g. comparison of effect estimates from different approaches, independent replication, bias analytic techniques, validation of instruments, simulations) | Sensitivity testing using Leave-one-out was used to assess outcome stability and to test whether single removal of variance affected the relationship between exposure and outcome. In addition, the MR Pleiotropy RESidual Sum and Outlier (MR-PRESSO) was used to detect outliers, and if outlying SNPs existed, they were excluded and reanalyzed, and the MR-Egger intercept was used to detect horizontal polytropy, and horizontal pleiotropy existed at P< 0.05. Heterogeneity among instrumental variable SNPs was tested using Cochran's Q test, when the Cochran Q test value of Q-Q was P> 0.05, it indicated the absence of heterogeneity, suggesting that there was no potential for horizontal polytropy to be examined and provided strong support for the IVW model. |
| 9 | **Software and pre-registration** |  |  |
|  | a) | Name statistical software and package(s), including version and settings used | R software version 4.3.2 was used for all analyses, and the TSMR package had been utilized. |
|  | b) | State whether the study protocol and details were pre-registered (as well as when and where) | Yes |
|  | **RESULTS** |  |  |
| 10 | **Descriptive data** |  |  |
|  | a) | Report the numbers of individuals at each stage of included studies and reasons for exclusion. Consider use of a flow diagram | The flow diagram is shown in Figure 2. |
|  | b) | Report summary statistics for phenotypic exposure(s), outcome(s), and other relevant variables (e.g. means, SDs, proportions) | In this study, using UA as an exposure factor, 233 SNPs were extracted as IVs after screening at P< 5.0×10-8 and excluding the chain imbalance. Strong instrumental variables were selected based on an F-value >30, resulting in the removal of 119 SNPs with F-value below 30. There was no possibility of weak instrumental variable bias. Subsequently, 55 SNPs that were associated with confounding factors were removed. Finally, a total of 59 genome-wide SNPs that were closely related to MI were selected as instrumental variables , and the MR-PRESSO method revealed no outliers. There is no horizontal multiplicity in the MR-Egger intercept test, and the findings are reliable. All detailed SNP information is provided in appendix S1 to S6. |
|  | c) | If the data sources include meta-analyses of previous studies, provide the assessments of heterogeneity across these studies | Inapplicable. |
|  | d) | For two-sample MR:  i.  Provide justification of the similarity of the genetic variant-exposure associations between the exposure and outcome samples  ii.  Provide information on the number of individuals who overlap between the exposure and outcome studies | The IVW method showed that UA is a risk factor for MI (OR = 1.333, 95% CI: 1.079-1.647). The MR Egger method calculated an OR of 1.616 (95% CI: 1.098-2.378), the Weighted Median method calculated an OR of 1.611 (95% CI: 1.214-2.138), and the Weighted Mode method calculated an OR of 1.759 (95% CI: 1.285-2.409). All P-values were less than 0.05, as shown in Table 3. |
| 11 | **Main results** |  |  |
|  | a) | Report the associations between genetic variant and exposure, and between genetic variant and outcome, preferably on an interpretable scale | UA is an independent risk factor for MI. |
|  | b) | Report MR estimates of the relationship between exposure and outcome, and the measures of uncertainty from the MR analysis, on an interpretable scale, such as odds ratio or relative risk per SD difference | The IVW method showed that UA is a risk factor for MI (OR = 1.333, 95% CI: 1.079-1.647). |
|  | c) | If relevant, consider translating estimates of relative risk into absolute risk for a meaningful time period | OR = 1.333, 95% CI: 1.079-1.647 |
|  | d) | Consider plots to visualize results (e.g. forest plot, scatterplot of associations between genetic variants and outcome versus between genetic variants and exposure) | In this study, the IVW method is considered the primary result (Figure 3a). Additionally, the funnel plot demonstrates a symmetrical distribution of data points, suggesting a low potential for underlying bias in the results obtained using the 59 SNPs as instrumental variables (Figure 3b). This indicates that the results are stable and reliable. |
| 12 | **Assessment of assumptions** |  |  |
|  | a) | Report the assessment of the validity of the assumptions | The MR Egger method calculated an OR of 1.616 (95% CI: 1.098-2.378), the Weighted Median method calculated an OR of 1.611 (95% CI: 1.214-2.138), and the Weighted Mode method calculated an OR of 1.759 (95% CI: 1.285-2.409). All P-values were less than 0.05, as shown in Table 3. |
|  | b) | Report any additional statistics (e.g., assessments of heterogeneity across genetic variants, such as *I^2^*, Q statistic or E-value) | Heterogeneity analysis of SNPs showed IVW Q=84.646, P=0.013,MR Egger Q=82.681, P=0.015.However, the heterogeneity was small with I^2^ =31.5%, which was negligible by choosing the random effects model of IVW to correct for it in this study. |
| 13 | **Sensitivity analyses and additional analyses** |  |  |
|  | a) | Report any sensitivity analyses to assess the robustness of the main results to violations of the assumptions | the MR-PRESSO method revealed no outliers. There is no horizontal multiplicity in the MR-Egger intercept test, and the findings are reliable. |
|  | b) | Report results from other sensitivity analyses or additional analyses | The examination of gene-level pleiotropy was conducted using the MR-Egger regression analysis, and the intercept term was -0.007 (P=0.249), with P> 0.05 indicating that there was no level pleiotropy. |
|  | c) | Report any assessment of direction of causal relationship (e.g., bidirectional MR) | MI was the exposure factor and UA was the outcome variable in reverse TSMR. A total of 11 SNPs were evaluated and found to be IVs, all with F-values> 10 (appendix S5). The horizontal pleiotropy test (Egger's intercept = -0.002, P=0.496) (Table 6) found no evidence of horizontal pleiotropy. The MR findings did not show a link between genetic vulnerability to MI and elevated UA levels (IVW, OR: 1.001, 95% CI: 0.989-1.012, P=0.922). Other techniques' results were MR-Egger (OR: 1.012, 95% CI: 0.978-1.048, P=0.501), weighted median (OR: 0.998, 95% CI: 0.986-1.009, P=0.705) and weighted mode (OR: 0.998, 95% CI: 0.985-1.010, P=0.737) (Table 5, appendix D3 and D4). Among the results of the heterogeneity test, MR-Egger regression showed relatively small heterogeneity (Cochran's Q=17.47, P=0.042), and relatively small heterogeneity among IVs was also found for IVW (Cochran's Q=18.45, P=0.048) (Table 6). The MR-PRESSO showed that the Global Test had no horizontal polytropy (Global Test RSSobs=20.87, P=0.073) and no outliers were observed. |
|  | d) | When relevant, report and compare with estimates from non-MR analyses | All these results indicated that UA plays an important role in MI. And the sensitivity analysis showed that the results of MR analysis were reliable. |
|  | e) | Consider additional plots to visualize results (e.g., leave-one-out analyses) | The "leave-one-out" method and the forest plot are shown in the appendix D5 and D6. |
|  | **DISCUSSION** |  |  |
| 14 | **Key results** | Summarize key results with reference to study objectives | Genetically predicted high blood UA levels were associated with a high risk of developing MI (IVW, OR=1.333, 95% CI:1.079-1.647, P=0.008), and inverse MR analysis did not demonstrate a causal relationship between MI on elevated UA levels (IVW, OR: 1.001,95% CI: 0.989-1.012, P=0.922 ). |
| 15 | **Limitations** | Discuss limitations of the study, taking into account the validity of the IV assumptions, other sources of potential bias, and imprecision. Discuss both direction and magnitude of any potential bias and any efforts to address them | Further stratified analyses could not be performed to explore the relationship between UA levels and different types of MI. In addition, the sensitivity analyses' results in the MR analysis were inconsistent, making it unable to fully rule out the effect of possibly confounding SNPs. Finally, the cross-sectional study and MR analysis data were not from the same sample. |
| 16 | **Interpretation** |  |  |
|  | a) | Meaning: Give a cautious overall interpretation of results in the context of their limitations and in comparison with other studies | The data on MI were obtained from questionnaires, and the type of MI was not specified, thus further stratified analyses could not be performed to explore the relationship between UA levels and different types of MI. In addition, the sensitivity analyses' results in the MR analysis were inconsistent, making it unable to fully rule out the effect of possibly confounding SNPs. Finally, the cross-sectional study and MR analysis data were not from the same sample; the study population for the data from NHANES was American, whereas the MR analysis population was participants of European ancestry, which makes our results regionally limited. These need to be investigated in more studies in the future. |
|  | b) | Mechanism: Discuss underlying biological mechanisms that could drive a potential causal relationship between the investigated exposure and the outcome, and whether the gene-environment equivalence assumption is reasonable. Use causal language carefully, clarifying that IV estimates may provide causal effects only under certain assumptions | The exact mechanism of the association between UA and MI has not been fully clarified, and current studies generally agree(39) that elevated UA has the effect of promoting oxidative stress, inflammation, and endothelial dysfunction in endothelial cells, which makes vascular smooth muscle cells proliferate and vasoconstriction, and exacerbates tissue hypoxia, and is an important pathologic mechanism by which elevated UA affects the occurrence of MI. Among them, inflammation plays a major role in UA-induced cardiomyocyte injury, which is closely related to the activation of the NOD-like receptor pyrin domain-containing protein 3 (NLRP3) inflammasome. UA was reported to induce ROS production through the activation of NLRP3 inflammasome(40). Subsequently, an experimental animal study demonstrated(6) that myocardial ischemia-reperfusion injury was aggravated through the ROS/NLRP3 inflammasome-mediated pyroptosis pathway. Another in vivo in vitro experiment further demonstrated(7) that high concentrations of UA may induce cardiomyocyte injury through activation of NLRP3 and ROS/TRPM2 channels/Ca2+. In addition, through a variety of methods, including blocking L-arginine absorption and stimulating L-arginine breakdown by arginase, UA can result in lower NO production and bioavailability(41). Meanwhile, the inflammatory response to tissue hypoxia during MI induces XO enzymes, which increase XO activity thereby increasing UA levels and ROS, promoting oxidative stress and leading to endothelial dysfunction(42). It has also been found(43) that elevated UA levels induce endothelial dysfunction through mitochondrial calcium overload mediated by mitochondrial Na+/Ca2+ exchangers. |
|  | c) | Clinical relevance: Discuss whether the results have clinical or public policy relevance, and to what extent they inform effect sizes of possible interventions | In conclusion, our cross-sectional study supports that UA is an independent risk factor for MI. Despite potential uncontrolled confounding factors that may affect observational results, by incorporating further TSMR analysis, we have discovered a causal link between increased UA levels and MI, increasing the risk of MI. These findings need to be confirmed by additional research, and the underlying mechanisms require further exploration. Future research on the function of UA in the prevention and treatment of MI may use these findings as a reference. |
| 17 | **Generalizability** | Discuss the generalizability of the study results (a) to other populations, (b) across other exposure periods/timings, and (c) across other levels of exposure | The use of two-way TSMR analysis addresses the effects of measurement bias, nonresponse bias, and recall bias in observational studies and avoids the influence of reverse causality on the results, further illustrating the causal relationship between UA levels and MI. Thus, our study is of fundamental importance in that it attempts to strengthen our comprehension of the causal relationship between UA and MI in a genomic context. In addition there are some limitations of this study. |
|  | **OTHER INFORMATION** |  |  |
| 18 | **Funding** | Describe sources of funding and the role of funders in the present study and, if applicable, sources of funding for the databases and original study or studies on which the present study is based | No funding. |
| 19 | **Data and data sharing** | Provide the data used to perform all analyses or report where and how the data can be accessed, and reference these sources in the article. Provide the statistical code needed to reproduce the results in the article, or report whether the code is publicly accessible and if so, where | The original contributions presented in the study are included in the article/[Supplementary Material](https://www.ncbi.nlm.nih.gov/pmc/articles/PMC10664243/#SM1). Further inquiries can be directed to the corresponding author. |
| 20 | **Conflicts of Interest** | All authors should declare all potential conflicts of interest | The authors declare that the research was conducted in the absence of any commercial or financial relationships that could be construed as a potential conflict of interest. |

This checklist is copyrighted by the Equator Network under the Creative Commons Attribution 3.0 Unported (CC BY 3.0) license.

1. Skrivankova VW, Richmond RC, Woolf BAR, Yarmolinsky J, Davies NM, Swanson SA, et al. Strengthening the Reporting of Observational Studies in Epidemiology using Mendelian Randomization (STROBE-MR) Statement. JAMA. 2021;under review.

2. Skrivankova VW, Richmond RC, Woolf BAR, Davies NM, Swanson SA, VanderWeele TJ, et al. Strengthening the Reporting of Observational Studies in Epidemiology using Mendelian Randomisation (STROBE-MR): Explanation and Elaboration. BMJ. 2021;375:n2233.
